# Interactive effects of precipitation and nitrogen enrichment on multi-trophic dynamics in plant-arthropod communities

**DOI:** 10.1371/journal.pone.0201219

**Published:** 2018-08-02

**Authors:** Kaitlin A. Griffith, Joshua B. Grinath

**Affiliations:** 1 Department of Biological Science, Florida State University, Tallahassee, Florida, United States of America; 2 Department of Biology, Middle Tennessee State University, Murfreesboro, Tennessee, United States of America; Institut Sophia Agrobiotech, FRANCE

## Abstract

Patterns of precipitation and nitrogen (N) deposition are changing in ecosystems worldwide. Simultaneous increases in precipitation and N deposition can relieve co-limiting soil resource conditions for plants and result in synergistic plant responses, which may affect animals and plant responses to higher trophic levels. However, the potential for synergistic effects of precipitation and N deposition on animals and plant responses to herbivores and predators (via trophic cascades) is unclear. We investigated the influence of precipitation and N enrichment on ecological dynamics across three trophic levels, hypothesizing that herbivores and plants would exhibit synergistic responses to the combined influence of precipitation, N amendments and predators. To test this, we conducted a field experiment with arthropods on two model plant species, *Nicotiana tabacum* and *Nicotiana rustica*. First, we characterized the plant-arthropod assemblages, finding that *N*. *tabacum* hosted greater abundances of caterpillars, while *N*. *rustica* hosted more sap-sucking herbivores. Next, we evaluated the effects of rainwater, soil N, and predatory spider manipulations for both plant-arthropod assemblages. On *N*. *tabacum*, water and N availability had an interactive effect on caterpillars, where caterpillars were most abundant with rainwater additions and least abundant when both rainwater and N were added. For *N*. *rustica*, foliar chemistry had a synergistic response to all three experimental factors. Compared to spider-absent conditions, leaf N concentration increased and C/N decreased when spiders were present, but this response only occurred under high water and N availability. Spiders indirectly altered plant chemistry via a facilitative effect of spiders on sap-sucking herbivores, potentially due to intra-guild predation, and a positive effect of sap-suckers on foliar N concentration. Our study suggests that predictions of the ecological impacts of altered precipitation and N deposition may need to account for the effects of resource co-limitation on dynamics across trophic levels.

## Introduction

Multiple factors are causing changes in Earth’s ecosystems [1], and we are coming to understand how these drivers of global change interact [2–5]. In particular, changes in precipitation and nitrogen (N) deposition are currently occurring in terrestrial ecosystems worldwide and further changes are expected [6–8]. It is becoming clear that shifts in precipitation and N deposition often have interactive effects on plants [9, 10] because plants are commonly co-limited by water and N supply [11, 12]. Under co-limitation, plant responses to single resources are constrained by the availability of other resources, and plant responses to multiple resources differ from those expected from single resource additions. For instance, Harpole and co-authors [9] found that primary production had a synergistic, or “super-additive”, response to increased precipitation and N deposition: plant production was greater than the additive expectation based on plant responses to water and N alone. Further studies suggest that this super-additive plant response may be widespread, especially in arid and semi-arid grasslands [10], and that precipitation and N deposition can have other interactive effects on plants, such as by altering plant defenses against herbivores [13].

**Fig 1:**
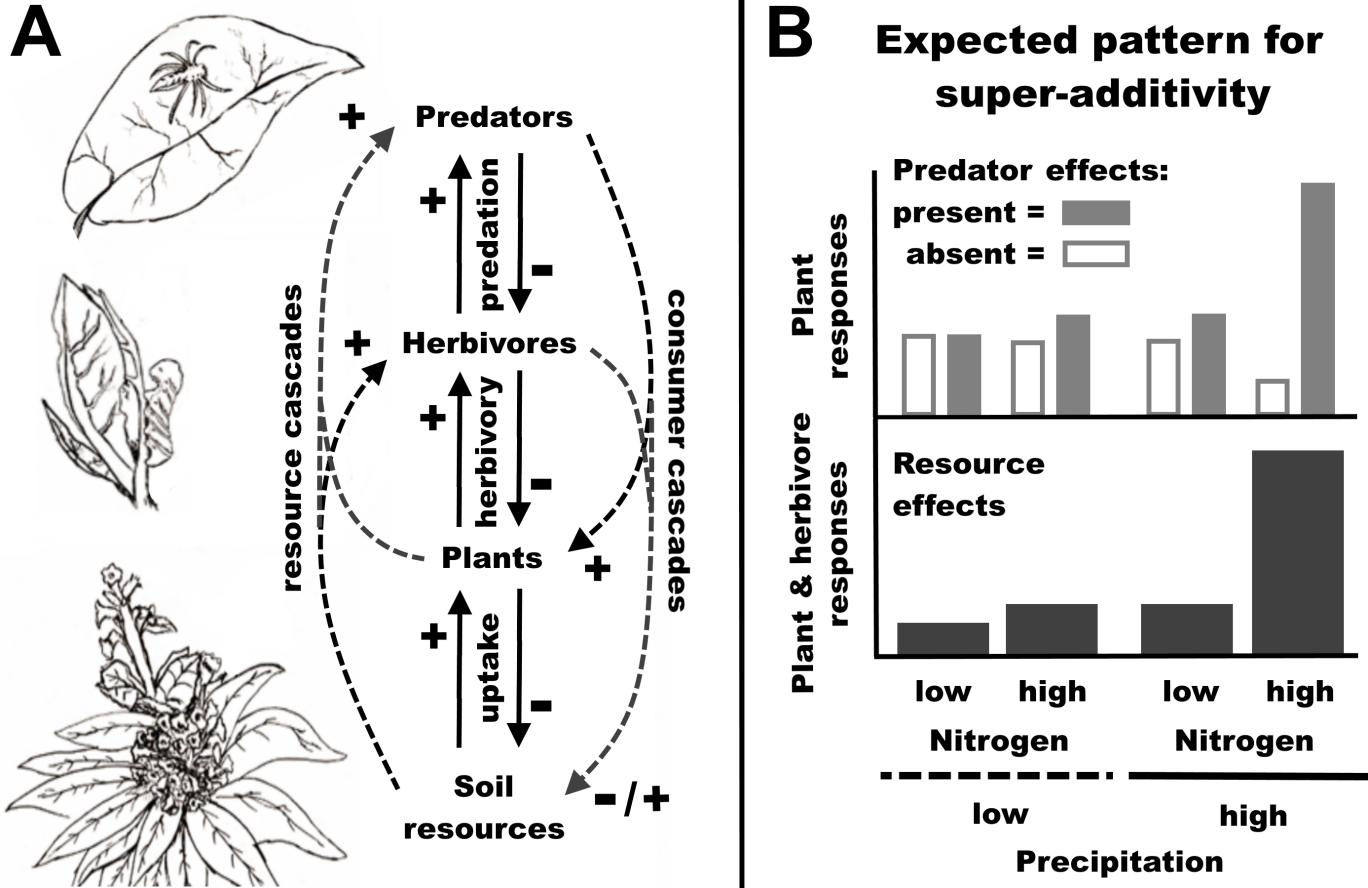
**Diagram of interactions between soil resources, plants, herbivores and predators (A), and expected patterns of super-additive response when herbivores occur on high quality host plants (B).** In the diagram, consumer-resource interactions are indicated by reciprocal solid arrows, with the type of interaction provided between the arrows and the sign of the effects provided near each arrowhead. Soil resources and organisms can have indirect effects across this chain of interactions, called cascades (dashed arrows). Cascades occur from resources to consumers (resource cascades) and from consumers to resources (consumer cascades). The present study focuses on cascades from soil resources to herbivores and from predators to plants. In (B), we mapped our predictions for super-additive changes in plant and herbivore responses to greater precipitation and nitrogen deposition when plants are high in quality for herbivores. The bottom panel in (B) shows the effects of resources on plants and herbivores, while the top panel depicts indirect predator effects on plants. Predator effects on herbivores (not shown) are expected to be the opposite of predator effects on plants. These hypothetical results depict positive super-additive responses; negative super-additive responses would show the reverse pattern, with relatively large responses at low resource levels and with single resource additions and very small responses when both resources are added.

Synergistic plant responses to water and N availability can have repercussions at higher trophic levels, but studies have found inconsistent animal responses to combined changes in precipitation and N deposition [14–21] and these dynamics are not well understood. Here, we refer to the indirect effects of resources on higher trophic levels (i.e. bottom-up effects) as ‘resource cascades’ (Fig 1A). These effects are in contrast to the indirect effects of consumers on lower trophic levels (i.e. top-down effects) [22], commonly called ‘trophic cascades’ [23–25], but which we refer to as ‘consumer cascades’ in explicit reference to the causal agent. Resource and consumer cascades can interact [22] and the strength of consumer cascades is often affected by resource availability for plants [26]. However, at present it is unclear how simultaneous changes in precipitation and N deposition influence plant responses within consumer cascades [20].

Precipitation can impact plant relationships with higher trophic levels by affecting plant water content. When plants are severely water stressed, plant proteins can become hydrolyzed and increase amino acid availability for herbivores, but low plant turgor inhibits herbivore access to these nutrients [27, 28]. As rainfall increases and begins to relieve plant water stress, these nutrients become more available to herbivores. When precipitation increases further, plants no longer become stressed by water availability and have operational protein metabolism, resulting in lower nutrient availability for herbivores as amino acids become diluted and incorporated into proteins [27, 28]. Water stress can also affect plant resistance to herbivores; ecological theory predicts that concentrations of carbon-based defensive compounds increase with water stress due to photosynthetic production in excess of growth demands [29]. Alternatively, concentrations of N-based defensive compounds have been shown to both increase and decrease with plant water stress [19, 30]. In addition to these changes in plant quality for herbivores, precipitation generally increases plant biomass production [31, 32], resulting in greater biomass of higher trophic levels [33–35]. As the number of predators increase, these dynamics often result in stronger cascades of effects on primary producers, but not always [26].

Similar to precipitation, N deposition also affects plant production, plant quality for herbivores, and the strength of consumer cascades [36]. Herbivory frequently intensifies as plant quality for herbivores is enhanced by N deposition [37, 38], which indirectly benefits predators that respond to greater prey quality and quantity [39, 40]. These fertilization effects can cause consumer cascades to intensify [26, 41], but N deposition can also result in weak consumer cascades by increasing plant resistance (tolerance and defenses) to herbivores and diminishing the effects that consumers can have on plants [42–44]. With N enrichment, theory predicts that plants with nitrogenous anti-herbivore defenses will have greater resources to devote to constitutive defenses [29]. A plant’s ability to induce defenses after herbivore damage depends on N availability as well [45–47]. Altogether, responses of herbivores and consumer cascades to N deposition, as well as precipitation, are contingent on how plants allocate resources to growth, reproduction and defense.

While much is known about the separate effects of precipitation and N deposition on multi-trophic dynamics, much less is known about the interactive effects of these global change factors. Of the handful of studies evaluating the combined influence of altered precipitation and N deposition across trophic levels, most have found interactive effects. Lightfoot and Whitford [14] found that greater rainfall and N availability had a synergistic effect on arthropod abundances on creosotebush (*Larrea tridentata*) that was primarily due to greater abundances of sap-sucking herbivores. Newingham and co-authors [15] also manipulated rainfall and N availability for creosotebush and found that mammalian herbivory increased only when both rainfall and N were enhanced, though chewing insect herbivory was unaffected. Testing the effects of N enrichment across a precipitation gradient, La Pierre and Smith [21] found that N additions increased leaf N and chewing herbivore abundances at the most mesic site and not at more xeric sites. However, the feeding rate of chewing herbivores simultaneously decreased with N enrichment at the mesic site; therefore, the N treatment did not alter the effect of invertebrate herbivores on plant biomass [21]. In a moss microcosm experiment, Kardol and co-authors [20] found that N addition suppressed nematode abundances, which were further suppressed by predators, but only when precipitation was infrequent. Studying tomato plants in growth chambers, Han and co-authors [16, 19] found that water and N availability had interactive effects on the development of leafminers. Yet, it is not always the case that precipitation and N deposition have interactive effects on multi-trophic dynamics. For instance, Han and co-authors [18, 19] did not find an interactive effect of water and N availability on phloem-feeding whiteflies or on the interactions between an omnivorous predator and its prey. Kardol et al. [20] also found that primary producer N content responded to predators in low N conditions but not when N was added, and these responses were unaffected by precipitation manipulations. In contrast, Lee and co-authors [17] found that plant-arthropod assemblages were sensitive to altered rainfall but not N availability. These variable responses are difficult to anticipate, but predictions for multi-trophic dynamics may be possible by considering resource co-limitation for plants and how different herbivore groups respond to changes in plants.

Variable responses of herbivores to precipitation and N deposition may occur because herbivores are not all similarly affected by changes in plants. Different herbivore groups and species derive nutrition from separate sources within plants, such as leaf-chewing and sap-sucking insects, and can perceive the quality of the same plant in different ways [27, 48]. For instance, though plant chemical defenses are generally detrimental for herbivores, small amounts of chemical defenses can stimulate feeding by resistant herbivores [49] and be beneficial for herbivores that coopt those chemicals for defense against predators [50]. Moreover, separate herbivore groups and species elicit different induced responses in plants [51] and mediate different pathways of effects that contribute to the net effect of predators on plants in consumer cascades [52–54]. Thus, to understand the impact of changes in precipitation and N deposition on animals and consumer cascades, we may often need to consider responses of separate herbivore groups in addition to the responses of multiple plant traits.

In this study, we asked whether greater precipitation (increasing from drought conditions) and N deposition have synergistic effects on herbivores via resource cascades and on plant responses in consumer cascades. We reasoned that if plants commonly have super-additive responses to precipitation and N availability [9, 10], then we may expect similar responses for herbivores and the effects of higher trophic levels on plants. We also expected that the direction of the response would depend on plant quality for different herbivore groups. We hypothesized that for herbivores on host plants on which they are most abundant (i.e. high quality hosts), greater rainwater and soil N availability result in positive super-additive responses characterized by low herbivore abundances at low resource levels and with single resource additions, and high herbivore abundances when both resources are added (Fig 1B). Predators generally benefit plants in consumer cascades across three trophic levels (Fig 1A [23]), and we expected that these benefits would depend on resource co-limitation for plants. We hypothesized that when predators suppress herbivores feeding on preferred hosts, plants have small responses to consumer cascades at low resource levels and when resources are added singly, but a large response to consumer cascades when resources are added in tandem (Fig 1B). When herbivores occur on host plants on which they have relatively low abundance (i.e. low quality hosts), we hypothesized that higher precipitation and N deposition result in negative super-additive herbivore and plant responses (ie. the reverse pattern of response from Fig 1B). This antagonistic response may be expected if plant quality for herbivores decreases with greater resource availability for plants.

To test these hypotheses, we simulated conditions of altered precipitation and N availability in a field experiment and evaluated changes in resource cascades on herbivore abundances and consumer cascades on plant traits in two plant-arthropod systems. Plants in the genus *Nicotiana*, commonly known as tobacco, are model systems for studying plant-arthropod interactions [55–57], and we chose two species for our experiment: *N*. *rustica* and *N*. *tabacum* (wild and cultivated tobacco, respectively). These species both require large amounts of N to synthesize N-based anti-herbivore defenses, such as nicotine [58], but the expression of these defenses and other traits differ between the species [59, 60]. We expected that separate herbivore functional groups (chewing and sap-sucking insects) would have different preferences for these plant species and that herbivore abundances would respond positively to resource additions to preferred hosts and negatively for non-preferred hosts. To evaluate herbivore preferences for plant traits and how changes in plants affected herbivores, we measured plant size, reproduction and foliar stoichiometry. While we anticipated that all three traits would respond to precipitation and N, we expected that foliar C/N stoichiometry, representing pools of carbon (C) and N within plants [61], would be particularly responsive to the presence of predators. *Nicotiana* spp. induce N-rich defenses in response to herbivore damage and we expected predator suppression of herbivores to decrease the need for defenses and N assimilation by plants, thereby increasing foliar C/N [62]. First we determined differences in plant traits and herbivore abundances for the two tobacco species. Then we assessed plant and herbivore responses to precipitation, N, and spider manipulations. By investigating precipitation and N availability in a model system, this study offers unique insight into the effects of these global change drivers on ecological dynamics across trophic levels.

## Material and methods

### Study system

We conducted this research at Florida State University’s Biological Research Facility at Mission Road in Tallahassee, Florida (30° 27' 29.488" N, 84° 19' 58.879" W) in the summer of 2014. We planted the two tobacco species into a tilled research plot (10.5 x 33 m). This field was used for tobacco cultivation over 50 years ago, and many tobacco herbivores still occur in the area and could naturally colonize plants in our study. Caterpillars (Lepidoptera) were mostly the specialists tobacco budworms (*Heliothis virescens*) and tobacco hornworms (*Manduca sexta*); other chewing herbivores (grasshoppers: Orthoptera; beetles: Coleoptera) occurred rarely. Sap-sucking herbivores (Hemiptera) included stilt bugs (Berytidae), leafhoppers (Cicadellidae), stink bugs (Pentatomidae), and leaf-footed bugs (Coreidae). Sap-sucking herbivores were identified to family and their diet-breadths are unclear. Green lynx spiders (*Peucetia viridans*, Oxyopidae) naturally colonized the tobacco plants: these generalist, active-hunting predators consume many different groups of herbivores, notably caterpillars and sap-sucking herbivores [63, 64]. Lynx spiders can be common on tobacco plants and are known to consume tobacco budworms and other caterpillars on tobacco [63].

### Experimental design

We factorially crossed treatments of ambient (drought) and added precipitation, low and high soil N, and the presence of predaceous spiders in 80 experimental plots. For the precipitation and N treatments, our goal was to create limiting and non-limiting resource conditions for plants to evaluate co-limitation. There were two plants per plot (one of each tobacco species) and each plot was 0.25 m^2^ for a total of 10 plots per treatment. Plants were grown in the greenhouse facility at Mission Road and were germinated from seed (*Nicotiana rustica* var. sacred cornplanter and *N*. *tabacum* var. celikhan) purchased from The Sustainable Seed Company (Chico, California). Plants were germinated in pots and as the plants grew, they were transplanted three times into larger pots (largest pot size 18.9 L). When the plants were six weeks old at the end of May, we buried the pots to soil level in the experimental field. Plants were randomly assigned to the northeast and southwest corner of each plot to minimize unintentional effects of light competition between plants (southern exposure had greater sunlight). Lower portions of the plants were touching, but the upper portions of plants tended to grow away from each other, further reducing the potential for light competition. We kept the plants in pots to minimize resource competition; however, the bottom of the pots had holes to allow root growth outside the containers, which minimized potential effects of being pot-bound [65].

We used conditions of ambient and added rainwater to manipulate precipitation. Precipitation was collected using a 10 m^2^ rain collector system that we adapted from the rain shelter design of Yahdjian and Sala [66]. Our design included seven corrugated plastic sheets (Tuftex 66 cm x 3.65 m) that shed water into two, 227 Liter Nalgene tanks. Weekly throughout June and July, we used a watering can to redistribute the collected rainwater equally among the 40 plots (total plot area = 10 m^2^) receiving double precipitation. The long term average precipitation for Tallahassee is 19.61 and 18.21 cm for June and July, respectively (years 1981–2010, source: U.S. Climate Data). Rainfall during the study was below average: 11.18 cm in June and 5.92 cm in July (frequency: 14 days and 10 days with ≥ 0.1 cm rainfall in June and July, respectively). Thus plants in ambient rainfall plots were exposed to water deficit conditions, while plants receiving double precipitation had water availability closer to average conditions. On several occasions during long periods without rainfall, all plants showed signs of wilting and were watered (1 cm well water applied to all treatments) to ensure survival through the end of the experiment. Ultimately, all plants were watered three times with well water and plants in the high rainfall treatment were watered another eight times with rainwater.

To simulate increased N deposition, we created conditions of low and high soil N within the pots. Plants in the low N treatment were not provided with N in addition to the amount present in the potting mix (Fafard #3). Nutrient content of the potting mix was sufficient for initial plant growth, but nutrient availability was expected to be limiting by field planting time. Plants in the high N treatment were supplied with a total of 2.6 g of ammonium nitrate fertilizer (34-0-0) spread over two equal applications (May 30 and June 12) beginning when pots were moved to the field. This level of N fertilization mimics the N-saturated environments that result from high levels of N deposition, and meets the uptake requirements for *N*. *tabacum* [67]. To focus on water and N co-limitation and to ensure primary nutrient limitation by N, every plant received 0.4 g of phosphate (0-45-0) and 3.0 g of potash (0-0-60) fertilizer on May 30 to meet uptake requirements [67] and remove the potential for primary nutrient limitation by phosphorus and potassium [68].

We manipulated predators by creating conditions with and without spiders. On June 4, we added two orb-weaving spiders to spider-present plots, sourcing the spiders from nearby vegetation. These web spiders did not remain on the experimental plants; however, a week later adequate numbers of lynx spiders were available to manipulate. We maintained lynx spider presence/absence in seven surveys over the next seven weeks. Lynx spiders were transferred to spider-present plots to maintain at least one spider/plot over this period, sourcing additional spiders from surrounding environments as needed. During the transfers, spiders were placed on the middle portion of the plants, but they were observed on all parts of the plants in subsequent surveys. Plots without spiders were maintained by manually removing spiders during the surveys. After a couple of weeks, transfers and removals were rarely needed because spiders remained within experimental plots, and were observed foraging on both plants within each plot.

### Data collection

To determine how precipitation and N enrichment influenced herbivores and consumer cascades, we measured plant trait responses and insect herbivore abundance and damage responses. Plant traits included aboveground dry mass (i.e. size), fruit production (i.e. reproduction), and foliar CN stoichiometry. All plant trait measurements were collected in the first several days of August, after nine weeks of field manipulations. We estimated aboveground dry mass by weighing plants that were cut at soil level and oven-dried at 65°C for five days. Fruit production is the total number of fruit per plant. To measure foliar C and N, we collected fresh leaf material from across the length of each plant, and immediately put these samples on ice in the field and then in a freezer in the lab before they were oven-dried at 65°C for five days. Next, we ground the leaves using mortar and pestle and measured foliar C and N concentrations with a Carlo Erba NA 1500 (Thermo Fisher Scientific, Waltham, MA) at the University of Florida’s Light Stable Isotope Mass Spectrometry Laboratory.

We measured herbivore responses as total herbivore abundance and as the abundances of the two most abundant taxonomic and functional groups, sap-sucking hemipterans and chewing caterpillars. These herbivore groups simultaneously colonized the experimental plants from surrounding environments. Herbivore abundances were counted in eight visual surveys from June 8 to July 23; then we calculated cumulative abundances to measure responses over the entire length of the experiment. We also measured chewing herbivore damage to leaves in the first several days of August. We visually assessed the proportion of leaf area damaged (using increments of 5% damage) on each of nine leaves sampled from across the length of the plant and averaged across all leaves to estimate damage over the whole plant. We did not measure predator abundances in response to rainwater and N manipulations because spider abundances were controlled and our focus was on the effects of these predators on herbivores and plants. We did not measure soil resource responses for similar reasons. Plants died in three of the experimental plots, and data from these plots were removed before analysis; thus, our dataset included responses for 77 individual plants per tobacco species. Data from this study are available in the Supporting Information (S1 Dataset).

### Data analysis

We analyzed our data with generalized linear models (GLMs) in the statistical software R v3.1.1 [69]. First, we determined differences between the tobacco species (including data from all experimental treatments), then we evaluated responses to the precipitation, N and spider manipulations (main effects and all interactions) for each tobacco species. All predictor variables were coded as binary factors. Gaussian GLMs were used for response data with approximately normal distributions, Poisson and Negative Binomial GLMs (‘MASS’ package) were used for count data, and Binomial GLMs were conducted for proportional data (i.e. chewing damage). We inspected the residuals of all models and occasionally removed outlying points to meet model assumptions [70]. This approach was used rather than transforming the data to meet model assumptions because synergistic responses must be assessed with untransformed data to avoid turning multiplicative relationships into additive relationships [71]. We performed Tukey contrasts (‘multcomp’ package) to evaluate post-hoc comparisons for significant statistical interactions in the GLMs. Type III sums of squares were used in all analyses (‘car’ package) to account for unequal numbers of observations among treatment groups, and effects were considered significant if *P* ≤ 0.05 and marginally significant if *P* ≤ 0.10.

To understand the GLM results better, we used two multi-group structural equation model (SEM) analyses to evaluate resource and consumer cascades across the ecological networks for *N*. *tabacum* and *N*. *rustica*. SEM is a multivariate statistical technique used to analyze the strength of direct and indirect effects across networks of variables [72, 73]. These networks are composed of directional hypotheses or ‘paths’ that can be standardized to units of standard deviation to evaluate relative effect sizes. Direct paths are calculated as bivariate regression coefficients or as partial regression coefficients, depending on whether or not the variables are connected by one or more directed pathways, respectively. Indirect effects are calculated as the product of the coefficients along a compound path, and net (total) effects are the sum of all direct and indirect effects. Unresolved, non-directional effects can also be included in SEMs and are measured as partial correlations. See the references herein for further details of SEM analyses [53, 54, 72, 73].

We conducted multi-group SEM analyses by applying the same model structures to data from *N*. *tabacum* and *N*. *rustica* separately. The resource cascade SEMs focused on the effects of the soil resource manipulations (rainwater and N) on plant traits (fruit production, aboveground mass, foliar C/N) and of plant traits on herbivore abundances (caterpillars and sap-suckers). The consumer cascade SEMs focused on the effects of the spider manipulation on herbivore abundances and of herbivores on plant traits (including foliar C and N concentrations and their direct effects on foliar C/N). We also considered unresolved, non-directional effects between caterpillars and sap-suckers and between plant traits. SEMs require at least five observations per directed path [73] and we had adequate replication to conduct analyses using data pooled across all soil resource and spider treatment levels for each plant species. However, we did not have enough replication to analyze each treatment group separately to assess statistical interactions in the GLMs with a multi-group SEM procedure. Nevertheless, the SEMs are useful for understanding general trends that can help to interpret the GLM results. Prior to analysis, we visually assessed scatterplots for all bivariate relationships among the variables and identified several extreme outlying observations whose removal was required to improve normality [72]. Of the original 77 observations for each plant species, two were removed for *N*. *tabacum* and three were removed for *N*. *rustica* (of which two were because of missing foliar chemistry data). The highest fruit production value was also removed from the *N*. *tabacum* dataset because it was an extreme outlier. The SEMs were conducted in AMOS 5.0.1 [74], using a χ^2^ lack-of-fit test to assess model fit. To understand which component pathways were responsible for net indirect effects (cascades), we measured standardized path coefficients and evaluated their contribution to total effects, considering paths significant when *P* ≤ 0.05 and marginally significant when *P* ≤ 0.10.

## Results

### Differences between plant-arthropod assemblages

**Fig 2:**
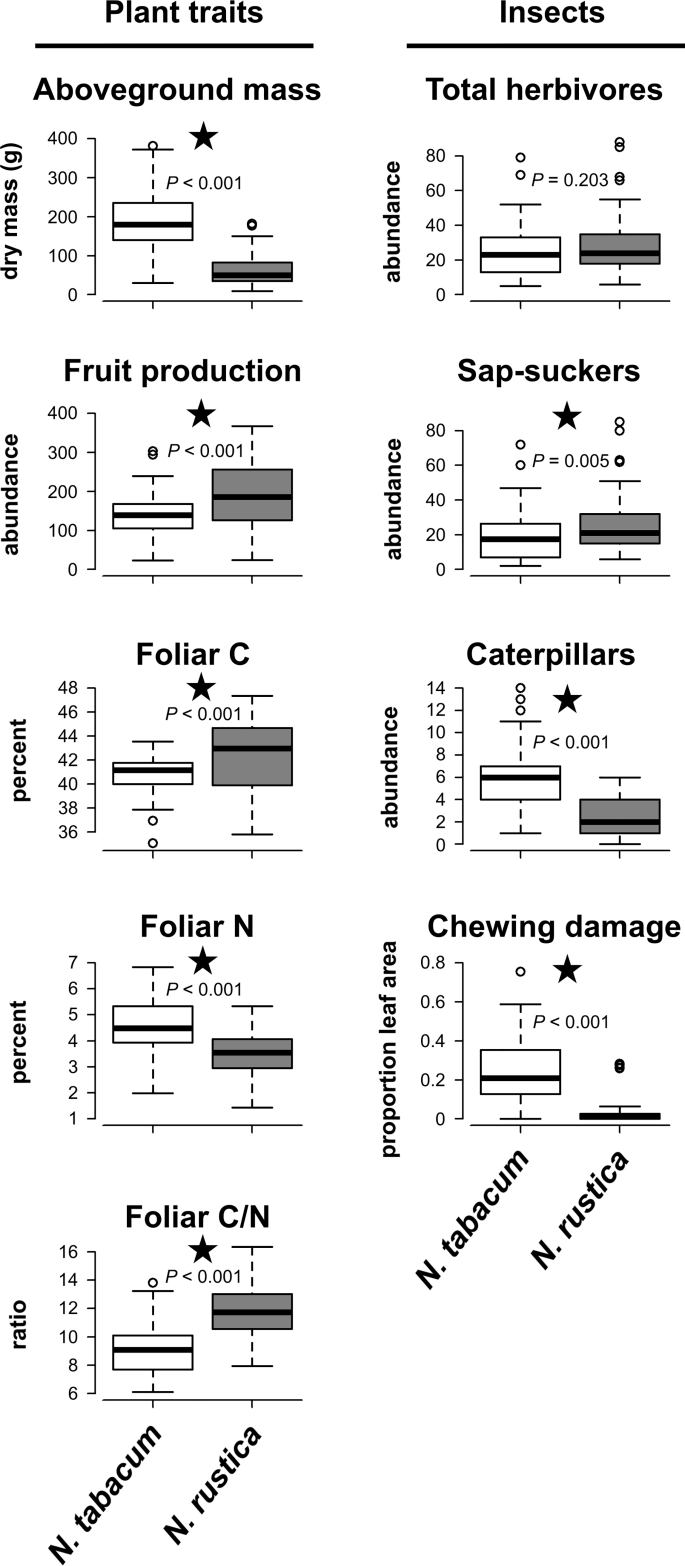
Differences in plant traits, insect herbivore abundances and chewing damage between the two tobacco species. Plant traits included aboveground biomass, fruit production, foliar C and N concentrations and foliar C/N. Insect responses included total herbivore abundance, the abundances of sap-sucking hemipterans and chewing caterpillars, and chewing damage to leaves. *P*-values are provided from generalized linear models (GLMs) and significant differences (*P* ≤ 0.05) between tobacco species are indicated with stars. All results are shown as boxplots.

The two tobacco species differed in all measured traits (Fig 2; S1 Table). *Nicotiana tabacum* had greater aboveground biomass (χ^2^ = 159.88, *P* < 0.001) and lower fruit production (χ^2^ = 15.18, *P* < 0.001) than *N*. *rustica*. Foliar N concentration was greater in *N*. *tabacum* (χ^2^ = 58.10, *P* < 0.001), while foliar C concentration was greater in *N*. *rustica* (χ^2^ = 17.29, *P* < 0.001). Therefore, foliar C/N was greater in *N*. *rustica* (χ^2^ = 88.95, *P* < 0.001).

Plant species had no effect on total abundances of insect herbivores (χ^2^ = 1.62, *P* = 0.203), but the abundances of herbivore functional groups were different between plants (Fig 2; S1 Table). While sap-sucking hemipterans comprised the majority of insects found on both plant species, these herbivores were more abundant on *N*. *rustica* (χ^2^ = 7.95, *P* = 0.005). In contrast, chewing caterpillars were more abundant on *N*. *tabacum* (χ^2^ = 98.70, *P* < 0.001) and chewing damage to leaves was also greater on these plants (χ^2^ = 17.70, *P* < 0.001). Other herbivores (grasshoppers and beetles) were rare and are not considered further.

### *Nicotiana tabacum* and associated herbivore responses

**Fig 3:**
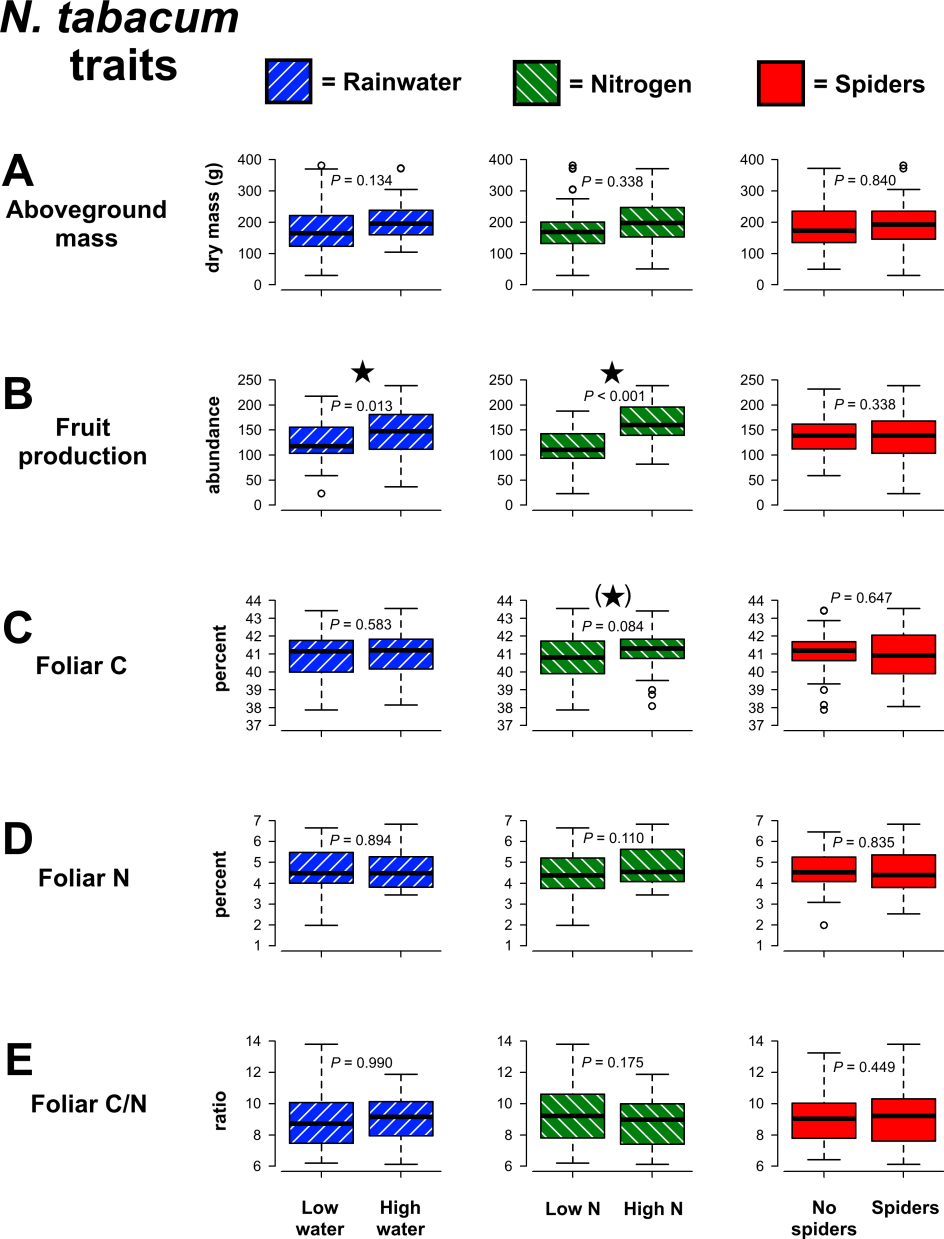
Effects of precipitation, nitrogen and predaceous spider manipulations on *Nicotiana tabacum* traits. Contrasts for the main effects are highlighted with colors corresponding with experimental treatments. *P*-values are from GLMs and significant differences (*P* ≤ 0.05) are indicated with stars, while marginally significant trends (*P* ≤ 0.10) are indicated by stars within parentheses. All results are shown as boxplots.

The experimental manipulations did not significantly affect the aboveground mass of *N*. *tabacum* (Fig 3A), but did have effects on fruit production (Fig 3B). Both greater rainwater (χ^2^ = 6.22, *P* = 0.013) and N availability (χ^2^ = 36.97, *P* < 0.001) resulted in higher fruit production, and these plant responses were independent of one another (i.e. additive). Foliar chemistry was not significantly affected by the water and N treatments (Fig 3C–3E), though there was a tendency for higher foliar C concentrations with greater N availability (χ^2^ = 2.98, *P* = 0.084). No *N*. *tabacum* traits were influenced by spiders or interactions between experimental manipulations (S2 Table).

**Fig 4:**
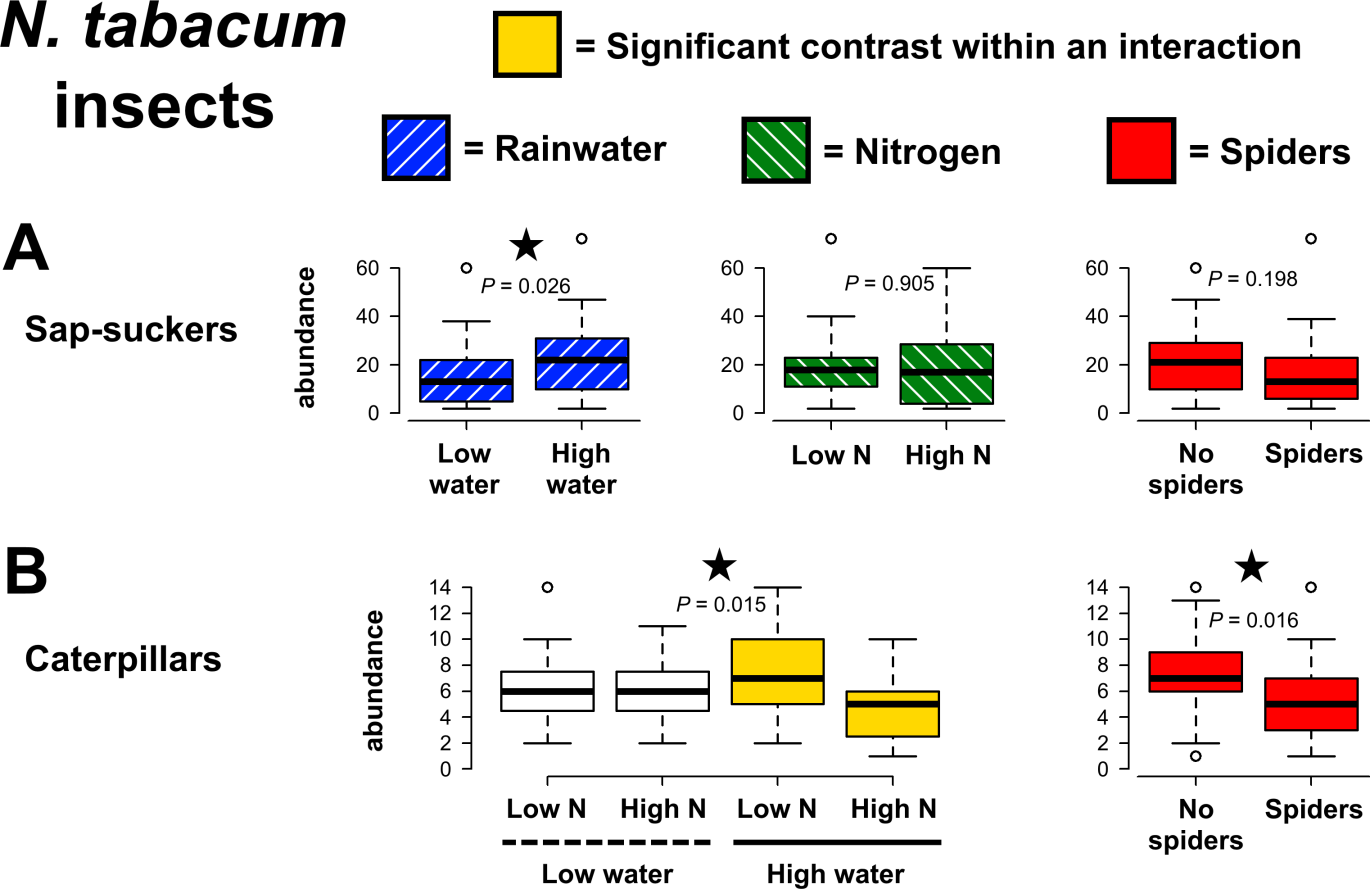
Effects of precipitation, nitrogen and predaceous spider manipulations on *Nicotiana tabacum* herbivores. Contrasts for main effects are highlighted with colors corresponding to treatments, except when a higher-order statistical interaction occurred. When this was the case, shading within the graph is used to indicate the two treatment groups that were significantly different from each other (Tukey post hoc: *P* ≤ 0.05). *P*-values are from GLMs and significant results (*P* ≤ 0.05) are indicated with stars.

Sap-sucker abundances on *N*. *tabacum* increased when rainwater availability was high (χ^2^ = 4.99, *P* = 0.026, Fig 4A), but did not respond to N additions, spiders, or interactive treatment effects (S2 Table). Conversely, rainwater and N availability had an interactive effect on caterpillar abundances (χ^2^ = 5.89, *P* = 0.015, Fig 4B). Greater abundances of caterpillars were found when rainwater alone was added, but lower abundances occurred when water and N were added together. Caterpillar abundances were also reduced by the presence of spiders (χ^2^ = 5.78, *P* = 0.016), yet this response was unaffected by rainwater and N amendment. No differences were found for chewing damage to plants across experimental treatments (S2 Table).

### *Nicotiana rustica* and associated herbivore responses

**Fig 5:**
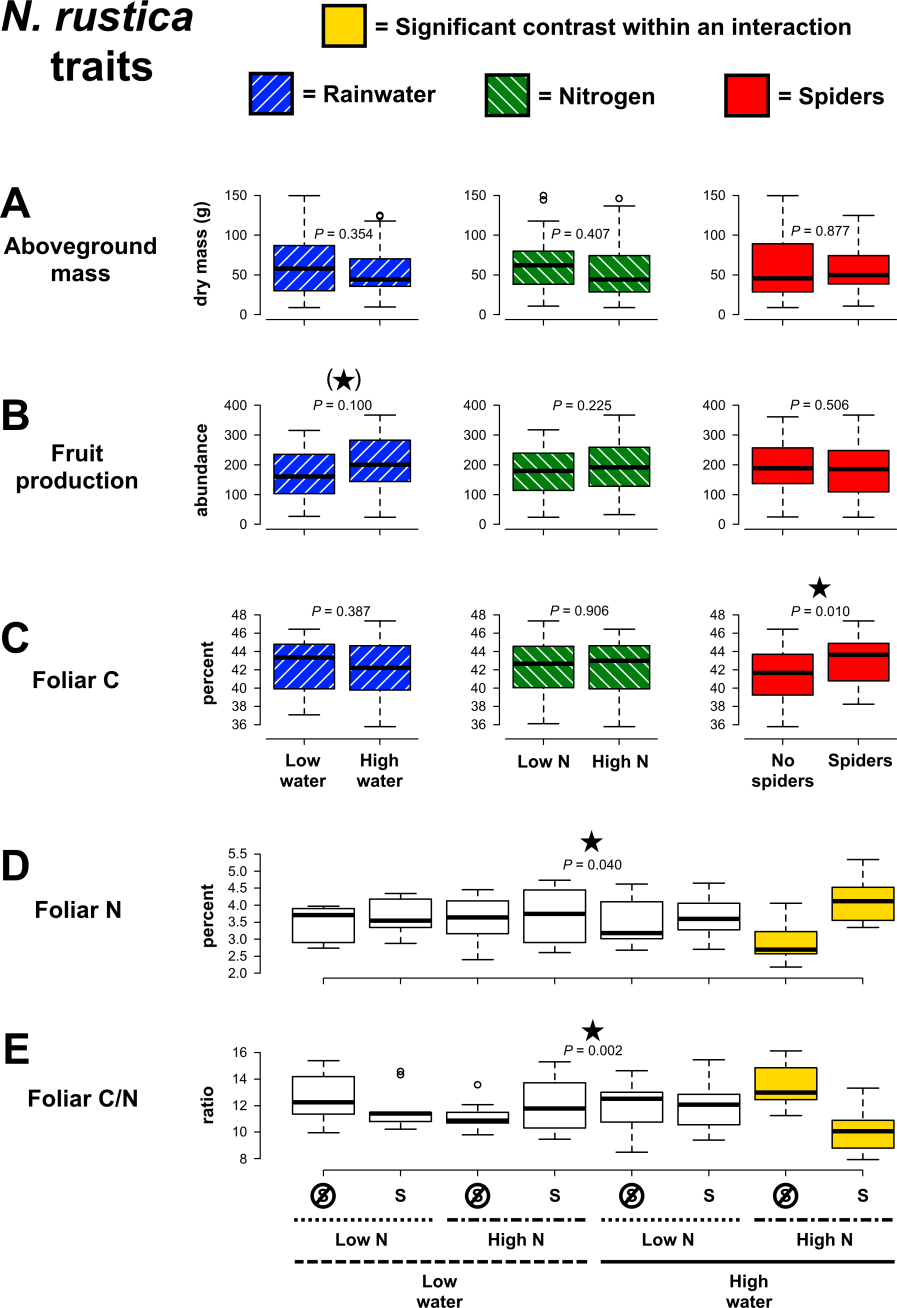
Effects of precipitation, nitrogen and spider manipulations on *Nicotiana rustica* traits. Contrasts for main effects are highlighted with colors corresponding to treatments, except when a higher-order statistical interaction occurred. When this was the case, shading within the graph is used to indicate the two treatment groups that were significantly different from each other (Tukey post hoc: *P* ≤ 0.05). *P*-values are from GLMs and significant results (*P* ≤ 0.05) are indicated with stars, while marginally significant trends (*P* ≤ 0.10) are indicated by stars within parentheses.

*Nicotiana rustica* aboveground mass and fruit production were not significantly affected by the experimental treatments (Fig 5A and 5B; S3 Table), though there was a trend of greater fruit production with higher rainwater (χ^2^ = 2.71, *P* = 0.100). However, foliar chemistry was significantly affected by the experimental treatments. While foliar C concentration was not significantly affected by precipitation, N enrichment or interactions between experimental treatments, foliar C concentration increased in response to spider presence (χ^2^ = 6.56, *P* = 0.010, Fig 5C). Both foliar N concentration (χ^2^ = 4.23, *P* = 0.040) and C/N ratio (χ^2^ = 9.55, *P* = 0.002) were influenced by three-way interactions between rainwater additions, N enrichment and spider presence (Fig 5D and 5E). Spider presence caused foliar N concentration to increase and foliar C/N to decrease, but only under high rainwater and soil N conditions.

**Fig 6:**
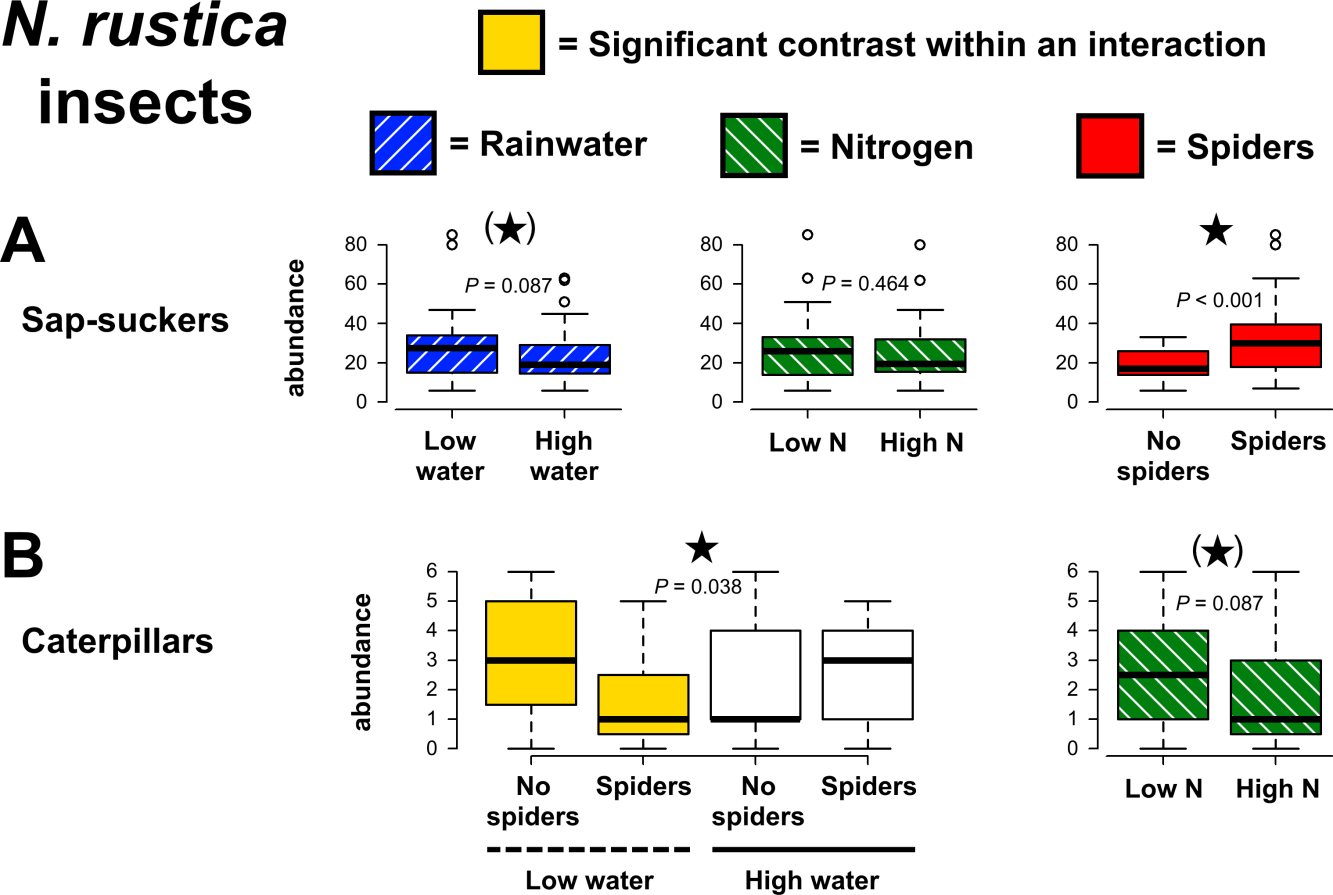
Effects of precipitation, nitrogen and predaceous spider manipulations on *Nicotiana rustica* herbivores. Contrasts for main effects are highlighted with colors corresponding to treatments, except when a higher-order statistical interaction occurred. When this was the case, shading within the graph is used to indicate the two treatment groups that were different from each other (Tukey post hoc: *P* ≤ 0.10). *P*-values are from GLMs and significant results (*P* ≤ 0.05) are indicated with stars, while marginally significant trends (*P* ≤ 0.10) are indicated by stars within parentheses.

Unexpectedly, sap-sucker abundances on *N*. *rustica* increased in the presence of spiders (χ^2^ = 20.10, *P* < 0.001, Fig 6A). Sap-suckers did not significantly respond to rainwater, N availability, or interactive effects of experimental treatments (S3 Table), but tended to be lower in abundance with greater rainwater (χ^2^ = 2.93, *P* = 0.087). In addition, spiders reduced caterpillar abundances, but only when rainwater availability was low (χ^2^ = 4.28, *P* = 0.038, Fig 6B). Caterpillar abundances also tended to be lower under N enrichment (χ^2^ = 2.93, *P* = 0.087). All other treatment effects on caterpillars and on chewing damage to plants were non-significant (S3 Table).

### Comparing pathways of resource and consumer cascades

**Fig 7:**
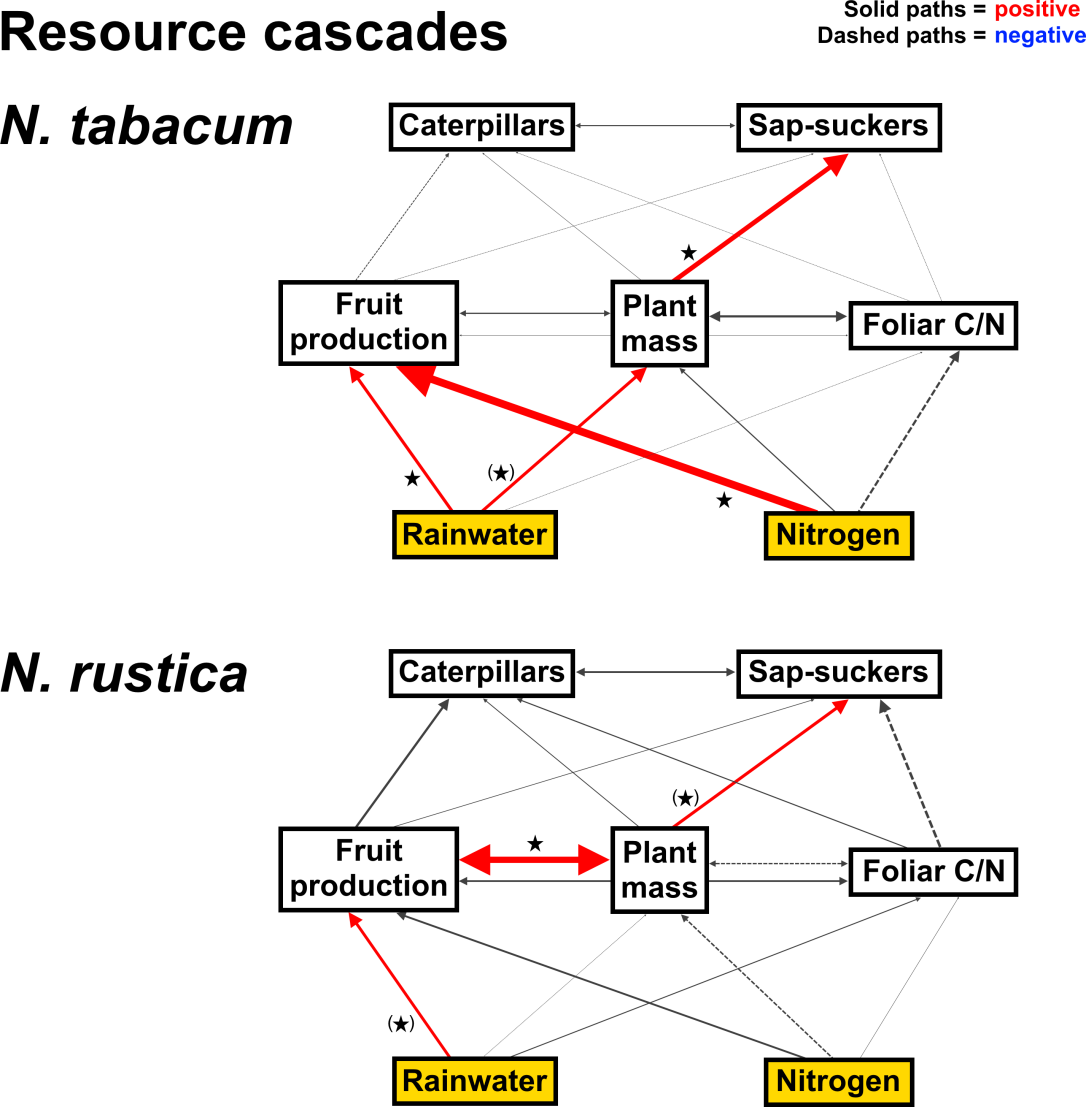
Path diagrams for the resource cascade multi-group structural equation model (SEM) analysis. Treatment (exogenous) variables are shown in yellow boxes and have direct effects (single-headed paths) on plant traits, which in turn have direct effects on herbivores. These dependent (endogenous) variables are depicted in white boxes. Double-headed paths are unresolved correlations. Solid paths are positive and dashed paths are negative, and the width of all paths is scaled by standardized effect size (paths < 0.025 are weighted as 0.025 to remain visible). Significant (*P* ≤ 0.05) and marginally significant (*P* ≤ 0.10) paths are shown in color (red: positive, blue: negative) and indicated by stars and parentheses around stars, respectively. Both models had adequate fit to the data (*N*. *tabacum*: *n* = 75, χ^2^ = 10.53, d.f. = 5, *P* = 0.062; *N*. *tabacum*: *n* = 74, χ^2^ = 7.82, d.f. = 5, *P* = 0.166).

The results of the resource cascade multi-group SEM analysis are depicted in Fig 7. Corresponding graphs of bivariate relationships between variables are in S1 Fig and S2 Fig for *N*. *tabacum* and *N*. *rustica*, respectively. Standardized and unstandardized effects, as well as standard errors and *P*-values, are provided in S4 Table and net effects are in S5 Table. This analysis indicates that resource cascades were stronger for the *N*. *tabacum*-herbivore network than that for *N*. *rustica*. Both rainwater and N additions increased fruit production by *N*. *tabacum*, whereas there was only the tendency for rainwater to increase fruit production by *N*. *rustica*. Rainwater additions also tended to increase the aboveground mass of *N*. *tabacum* and had a positive indirect effect on sap-sucker abundances through this effect on mass. Sap-sucker abundances tended to increase with the aboveground mass of *N*. *rustica* as well, but effects of soil resources on plant mass and herbivore abundances were unclear. Though we found evidence for a cascade from soil resources to caterpillars on *N*. *tabacum* in the GLM analysis, these effects were not evident in the SEM analysis.

**Fig 8:**
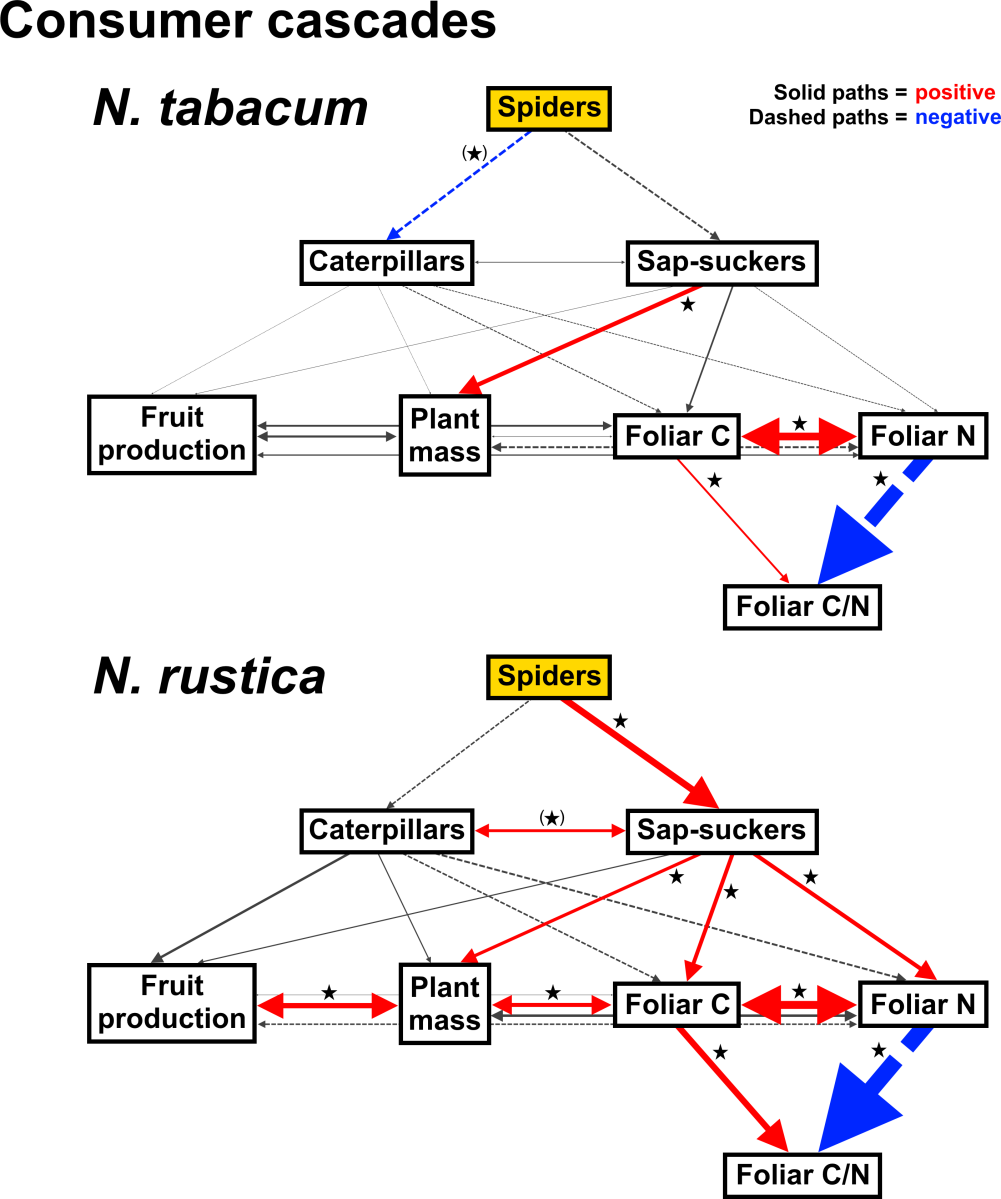
Path diagrams for the consumer cascade multi-group SEM analysis. Spider treatment (exogenous variable) is shown as a yellow box and has direct effects (single-headed paths) on herbivores, which in turn have direct effects on plant traits. Direct effects of foliar C and N concentrations on foliar C/N are also included to assess their relative contributions to this ratio. The dependent (endogenous) variables are depicted in white boxes; double-headed paths are unresolved correlations. Solid paths are positive and dashed paths are negative, and the width of all paths is scaled by standardized effect size (paths < 0.025 are weighted as 0.025 to remain visible). Significant (*P* ≤ 0.05) and marginally significant (*P* ≤ 0.10) paths are shown in color (red: positive, blue: negative) and indicated by stars and parentheses around stars, respectively. Both models had adequate fit to the data (*N*. *tabacum*: *n* = 75, χ^2^ = 9.84, d.f. = 9, *P* = 0.364; *N*. *tabacum*: *n* = 74, χ^2^ = 3.97, d.f. = 9, *P* = 0.914).

The consumer cascade multi-group SEM analysis is illustrated in Fig 8. Path estimates and net effects are in S6 Table and S7 Table, respectively. In contrast with above, this analysis indicates that consumer cascades were stronger in the *N*. *rustica* network than for *N*. *tabacum*. In the *N*. *rustica* network, spider presence positively affected sap-sucker abundances, and sap-suckers positively affected foliar C and N concentrations. Foliar N concentration had a greater effect on the C/N ratio than foliar C concentration, leading to a negative indirect effect of sap-suckers on foliar C/N and a negative net effect of spiders on foliar C/N. Foliar C and N concentrations were positively related, as were foliar C and plant mass, and fruit production and plant mass. Caterpillar and sap-sucker abundances tended to be positively correlated, suggesting that these herbivore groups did not strongly compete but preferred similar host plants. Sap-suckers also had a positive effect on plant mass, but the directionality of this effect may be questionable considering that we found evidence for the opposite effect in the resource cascade model. This path is significant in the consumer cascade model for *N*. *tabacum* too, as are all paths among the foliar chemistry variables. However, the *N*. *tabacum* network does not have significant pathways leading from spiders to plants. Spiders tended to decrease caterpillar abundances on *N*. *tabacum*, but caterpillars did not significantly affect plant traits in this network. Overall, this analysis indicates that spiders affected *N*. *rustica* foliar C/N via their interaction with sap-suckers, and that this cascade intensified when both rainwater and nitrogen were added (Fig 5E).

## Discussion

Our investigation indicates that precipitation and N deposition can have interactive effects on ecological dynamics across trophic levels. Specifically, we found that rainfall and N enrichment had a synergistic effect on plant chemistry responses to predators (*N*. *rustica*) and a different interactive effect on herbivore abundances (on *N*. *tabacum*). These interactive effects corresponded with the strength of consumer and resource forces in the ecological networks, where resource cascades were stronger for *N*. *tabacum* and consumer cascades were stronger for *N*. *rustica*. Together with previous studies [14–16, 19–21], our results suggest that changes in precipitation and N deposition may often have interactive effects on multi-trophic ecological dynamics.

Changes in multi-trophic dynamics are not generally taken into account when predicting plant responses to global change [75–77], even though feedbacks between plants and higher trophic levels can greatly influence plant communities and ecosystem functioning [22, 24]. In our study, co-limitation of rainwater and soil N availability constrained plant chemistry responses to herbivores and predators. Even small changes in plant chemistry can have large effects across the ecological community, altering plant fitness [55], competition [56], nutrient cycling [78], and plant quality for later-arriving herbivores [79]. Our results suggest that simultaneous increases in precipitation and N deposition relieve plants from co-limitation and lead to greater than expected changes in plant chemistry and other traits responding to soil resources and higher trophic levels. As climate and N deposition continue to change around the world, responses to N enrichment are likely to be constrained by drying conditions in many areas, while synergistic responses are likely to occur during episodic wet periods (ex. El Niño/La Niña) and some areas predicted to increase in precipitation [8]. Ecologists may often need to consider how resource co-limitation for plants affects multi-trophic dynamics to accurately predict plant and ecosystem responses to altered precipitation and N deposition.

The present study also demonstrates the importance of considering herbivore functional groups separately. We found that chewing and sap-sucking herbivores had different responses to rainwater and soil N supply in resource cascades and played different roles within the consumer cascade from spiders to plants. Furthermore, interactive effects of precipitation and N manipulations corresponded with herbivore functional groups on plant species on which they were most abundant: caterpillars on *N*. *tabacum* and sap-suckers on *N*. *rustica* (herbivores that mediated the cascade). These results suggest that the multi-trophic consequences of precipitation and N deposition are most likely to occur via herbivore groups feeding on plants perceived as high quality hosts, which we interpret as host preference. Even so, it may still be difficult to predict how herbivores will respond to changes in their preferred host plants.

Inconsistent with our predictions, caterpillars on their preferred host responded positively to rainwater additions, but negatively to concurrent rainwater and N additions. Conversely, the trends in caterpillar abundances on their non-preferred host, *N*. *rustica*, were more consistent with our expectations. On these plants, spiders had less of a suppressive effect on caterpillars under high rainwater availability and caterpillar abundances also tended to be lower on their non-preferred host plant when more N was available to plants. These negative responses suggest that plants may have had greater defenses against caterpillars when greater resources were available [58, 80], though evidence for this was not apparent from *N*. *tabacum* foliar CN chemistry, which was unresponsive to rainwater and N manipulations. We used a stoichiometric approach to understand how gross pools of C and N were altered by precipitation and N enrichment; however, this approach integrates plant chemistry across multiple metabolic functions [61] and can mask subtle changes in specific C and N-rich compounds. While we did not measure specific defensive compounds within *N*. *tabacum*, it is possible that relief from co-limiting resource conditions may have allowed these plants to synthesize defenses that were detrimental for caterpillars, but this requires additional study.

As expected, we found that plant responses within a consumer cascade were contingent on rainwater and soil N availability for plants, but the direction of this response was opposite that which we predicted. Spiders had an unexpected positive effect on sap-suckers, resulting in effects on *N*. *rustica* foliar chemistry. Though lynx spiders are predators of sap-sucking insects [63, 64], these spiders also prey on other predators, such as wasps that consume sap-sucking herbivores [81]. Such intraguild predation can create enemy-free space for herbivores and allow herbivores to increase in abundance [54, 82]. Enemy-free space for sap-suckers may have also been reinforced if spiders were satiated by feeding on caterpillars. Spiders reduced the abundances of caterpillars and we may have expected low caterpillar abundances to correlate with high sap-sucker abundances due to reduced competition and/or greater enemy-free space, but a negative link between caterpillar and sap-sucker abundances was not evident in the SEMs. Though intraguild predation is a plausible mechanism for the positive effect of spiders on sap-sucking insects, we do not have data on predators from our experiment, and this intriguing result also requires further study to elucidate. Similar to previous findings [63, 64, 81], intraguild predation may be likely to limit the effectiveness of lynx spiders as a biocontrol agent on cultivated tobacco and other economically important plant species.

Greater abundances of sap-sucking herbivores corresponded with higher concentrations of foliar N in *N*. *rustica*. Higher N concentration is consistent with our prediction that greater herbivore abundances induce plants to assimilate more N to synthesize N-rich defenses and that this response is constrained by N availability for plants [45, 46]. Proportionally, foliar N increased more than foliar C concentration, resulting in a lower C/N ratio as sap-sucker abundances increased. Moreover, induced defenses in tobacco suppress photosynthesis [83], which may further lead to lower plant C content and C/N ratios. However, greater sap-sucker abundances also corresponded with higher foliar C concentrations. Sap-suckers may have caused foliar C concentrations to increase by inducing the synthesis of C-based defenses or edible nutrient “sinks” within plants. For instance, a previous study with *N*. *tabacum* found that sap-sucking aphids attracted edible C-based compounds to leaves [84]. Sap-sucking herbivores can also alter plant N metabolism [85], and may have caused greater concentrations of edible N-based compounds in leaves, but the reliance of tobacco on induced nitrogenous defenses suggests that defensive chemistry is the more likely explanation for changes in foliar N concentration and C/N. Regardless of the mechanism of induction, it is clear that the synergistic foliar C/N response of *N*. *rustica* was dependent on the availability of both soil N and rainwater.

Our predictive framework for the effects of precipitation and N deposition on multi-trophic dynamics (Fig 1) was useful for anticipating synergistic responses consistent with resource co-limitation, but refinements are needed to obtain more accurate predictions. Our results suggest that the framework can be improved by considering the responses of specific plant metabolites and multiple predators. Additionally, though we used an additive experimental design, the conceptual model may be extended to continuous gradients of precipitation and N deposition, which could be assessed with response-surface experimental designs. Consideration of continuous gradients may reveal more detailed non-linear patterns, such as hump-shaped relationships in plant quality for herbivores across large precipitation gradients [27, 28].

Combinations of global change drivers other than precipitation and N deposition can have interactive effects across trophic levels as well [39, 40, 86–88], and our conceptual model may be adapted to understand the multi-trophic consequences of co-limitation in these contexts. For instance, atmospheric warming decreases soil moisture [8] and may thereby influence the fertilization effects of N deposition. In addition, N and phosphorus co-limit plant production in ecosystems around the world [71, 89, 90], and human activities are simultaneously increasing the availability of these nutrients, particularly in aquatic systems [91, 92]. Also, fossil fuel combustion is increasing CO_2_ availability for primary production [93], which in combination with nutrient enrichment can cause responses of primary producers indicative of co-limitation [94–96]. In all these cases, synergistic and other non-additive responses by primary producers are likely to influence higher trophic levels, potentially restructuring ecological networks and altering ecosystem functioning. To predict ecosystem responses to multiple drivers of global change, ecologists must form a deeper understanding of how resource co-limitation for primary producers affects dynamics across trophic levels.

## Supporting information

S1 Dataset Data for the research article “interactive effects of precipitation and nitrogen enrichment on multi-trophic dynamics in plant-arthropod communities” by Griffith and Grinath. (XLSX)

S1 Table Generalized linear model results for differences in traits and associated herbivores between the tobacco species. (PDF)

S2 Table Generalized linear model results for experimental effects on *Nicotiana tabacum* traits and associated herbivores. (PDF)

S3 Table Generalized linear model results for experimental effects on *Nicotiana rustica* traits and associated herbivores. (PDF)

S4 Table Standardized and unstandardized structural equation model results for resource cascades from nitrogen and rainwater additions to herbivores. (PDF)

S5 Table Matrices for standardized and unstandardized structural equation model total effects for resource cascades from nitrogen and rainwater additions to herbivores. (PDF)

S6 Table Standardized and unstandardized structural equation model results for the consumer cascade from spiders to plant traits. (PDF)

S7 Table Matrices for standardized and unstandardized structural equation model total effects for the consumer cascade from spiders to plant traits. (PDF)

S1 Fig Bivariate scatterplots for variables used in the *Nicotiana tabacum* structural equation models. (PDF)

S2 Fig Bivariate scatterplots for variables used in the *Nicotiana rustica* structural equation models. (PDF)
