# Supplementary material for: Interactive effects of precipitation and nitrogen enrichment on multi-trophic dynamics in plant-arthropod communities
Source: PLoS One. 2018 Aug 2;13(8):e0201219. doi: 10.1371/journal.pone.0201219 (PMC6072000; doi:10.1371/journal.pone.0201219)
Supplement: S1 Table — (PDF) [file pone.0201219.s002.pdf]

S1 Table. Generalized linear model results for differences in traits and associated herbivores between the tobacco species.

Dependent Variable	Distribution	N. Obs	χ^2	*P*
Aboveground plant mass (grams)	Gaussian	149	**159.880**	**<0.001**
Foliar chemistry				
C concentration	Gaussian	147	**17.290**	**<0.001**
N concentration	Gaussian	152	**58.102**	**<0.001**
CN Ratio	Gaussian	149	**88.947**	**<0.001**
Fruit abundance	Gaussian	154	**15.179**	**<0.001**
Herbivore abundances				
Total abundance	Negative binomial	152	1.623	0.203
Caterpillar abundance	Negative binomial	153	**98.699**	**<0.001**
Sap-sucker abundance	Negative binomial	150	**7.949**	**0.005**
Chewing damage (proportion)	Binomial	154	**17.696**	**<0.001**

Note: Significant results ($P \leq 0.05$) are shown in **bold**.
