# Supplementary material for: Interactive effects of precipitation and nitrogen enrichment on multi-trophic dynamics in plant-arthropod communities
Source: PLoS One. 2018 Aug 2;13(8):e0201219. doi: 10.1371/journal.pone.0201219 (PMC6072000; doi:10.1371/journal.pone.0201219)
Supplement: S2 Table — (PDF) [file pone.0201219.s003.pdf]

S2 Table. Generalized linear model results for experimental effects on *Nicotiana tabacum* traits and associated herbivores.

			Nitrogen		Water		Spiders				
Dependent Variable	Distribution	N. Obs	χ^2	*P*	χ^2	*P*	χ^2	*P*	Interactions	χ^2	*P*
Aboveground plant mass (grams)	Gaussian	72	0.917	0.338	2.241	0.134	0.041	0.840	N*W	0.283	0.594
									N*S	0.638	0.425
									W*S	0.544	0.461
									N*W*S	0.468	0.494
Foliar chemistry: C concentration	Gaussian	75	2.977	0.084	0.301	0.583	0.210	0.647	N*W	0.346	0.556
									N*S	0.479	0.489
									W*S	1.944	0.163
									N*W*S	2.986	0.084
N concentration	Gaussian	77	2.549	0.110	0.018	0.894	0.043	0.835	N*W	1.636	0.201
									N*S	0.051	0.822
									W*S	0.291	0.589
									N*W*S	1.741	0.187
CN ratio	Gaussian	76	1.844	0.175	<0.001	0.990	0.574	0.449	N*W	1.238	0.266
									N*S	0.324	0.569
									W*S	<0.001	0.984
									N*W*S	2.631	0.105
Fruit abundance	Gaussian	73	**36.973**	**<0.001**	**6.224**	**0.013**	0.919	0.338	N*W	0.775	0.379
									N*S	0.027	0.870
									W*S	1.071	0.301
									N*W*S	0.776	0.378
Herbivores: Caterpillar abundance	Poisson	76	**6.917**	**0.009**	0.697	0.404	**5.776**	**0.016**	N*W	**5.886**	**0.015**
									N*S	1.933	0.164
									W*S	0.265	0.607
									N*W*S	0.738	0.390
Sap-sucker abundance	Negative binomial	76	0.014	0.905	**4.987**	**0.026**	1.655	0.198	N*W	0.023	0.876
									N*S	0.377	0.539
									W*S	0.068	0.795
									N*W*S	0.403	0.525
Chewing damage (proportion)	Binomial	74	0.003	0.960	0.192	0.661	0.607	0.436	N*W	0.328	0.567
									N*S	0.319	0.572
									W*S	0.020	0.888
									N*W*S	0.090	0.764

Note: Significant results ($P \leq 0.05$) are shown in **bold**. N, W and S represent nitrogen, water and spider treatments, respectively.
