# Supplementary material for: Interactive effects of precipitation and nitrogen enrichment on multi-trophic dynamics in plant-arthropod communities
Source: PLoS One. 2018 Aug 2;13(8):e0201219. doi: 10.1371/journal.pone.0201219 (PMC6072000; doi:10.1371/journal.pone.0201219)
Supplement: S3 Table — (PDF) [file pone.0201219.s004.pdf]

S3 Table. Generalized linear model results for experimental effects on *Nicotiana rustica* traits and associated herbivores.

			Nitrogen		Water		Spiders				
**Dependent Variable**	**Distribution**	**N. Obs**	**χ^2**	***P***	**χ^2**	***P***	**χ^2**	***P***	**Interactions**	**χ^2**	***P***
Aboveground plant mass (grams)	Gaussian	75	0.687	0.407	0.860	0.354	0.024	0.877	N*W	0.378	0.539
									N*S	0.224	0.636
									W*S	0.446	0.504
									N*W*S	0.881	0.348
Foliar chemistry: C concentration	Gaussian	70	0.014	0.906	0.747	0.387	**6.560**	**0.010**	N*W	0.778	0.378
									N*S	0.052	0.820
									W*S	2.559	0.110
									N*W*S	0.916	0.339
N concentration	Gaussian	72	0.063	0.801	0.020	0.888	**9.025**	**0.003**	N*W	0.168	0.682
									N*S	2.576	0.109
									W*S	3.572	0.059
									N*W*S	**4.231**	**0.040**
CN ratio	Gaussian	71	0.535	0.465	0.003	0.960	**4.148**	**0.042**	N*W	0.232	0.630
									N*S	1.002	0.317
									W*S	**4.841**	**0.028**
									N*W*S	**9.549**	**0.002**
Fruit abundance	Gaussian	77	1.470	0.225	2.712	0.100	0.443	0.506	N*W	1.472	0.225
									N*S	2.425	0.119
									W*S	0.293	0.588
									N*W*S	0.058	0.810
Herbivores: Caterpillar abundance	Poisson	77	2.929	0.087	0.128	0.720	0.709	0.400	N*W	0.331	0.565
									N*S	0.447	0.504
									W*S	**4.284**	**0.038**
									N*W*S	3.656	0.056
Sap-sucker abundance	Negative binomial	74	0.536	0.464	2.933	0.087	**20.100**	**<0.001**	N*W	0.449	0.503
									N*S	0.309	0.578
									W*S	0.198	0.656
									N*W*S	1.698	0.193
Chewing damage (proportion)	Binomial	73	0.019	0.892	0.002	0.964	0.003	0.960	N*W	0.028	0.868
									N*S	0.006	0.936
									W*S	0.141	0.708
									N*W*S	0.022	0.883

Note: Significant results ($P \leq 0.05$) are shown in **bold**. N, W and S represent nitrogen, water and spider treatments, respectively.
