# Supplementary material for: Interactive effects of precipitation and nitrogen enrichment on multi-trophic dynamics in plant-arthropod communities
Source: PLoS One. 2018 Aug 2;13(8):e0201219. doi: 10.1371/journal.pone.0201219 (PMC6072000; doi:10.1371/journal.pone.0201219)
Supplement: S4 Table — (PDF) [file pone.0201219.s005.pdf]

S4 Table. Standardized and unstandardized structural equation model results for resource cascades from nitrogen and rainwater additions to herbivores on (A) *Nicotiana tabacum* and (B) *N. rustica*.

**A** **N. tabacum Variables** Independent >>>	Dependent	Unstandardized estimate	SE	*P*	Standardized estimate
**Direct Effect**					
Foliar C/N	Caterpillars	-0.007	0.215	0.974	-0.004
Foliar C/N	Sap-suckers	-0.214	0.907	0.814	-0.026
Fruit	Caterpillars	-0.004	0.008	0.630	-0.057
Fruit	Sap-suckers	-0.008	0.033	0.809	-0.027
Mass	Caterpillars	0.001	0.004	0.858	0.021
Mass	Sap-suckers	**0.045**	**0.016**	**0.006**	**0.309**
Nitrogen addition	Foliar C/N	-0.513	0.405	0.205	-0.146
Nitrogen addition	Fruit	**49.562**	**9.633**	**<0.001**	**0.505**
Nitrogen addition	Mass	15.064	22.150	0.496	0.077
Rainwater addition	Foliar C/N	-0.003	0.405	0.995	-0.001
Rainwater addition	Fruit	**20.031**	**9.633**	**0.038**	**0.204**
Rainwater addition	Mass	**39.376**	**22.150**	**0.075**	**0.202**
**Unresolved Covariance/Correlation**					
Caterpillars	Sap-suckers	3.066	5.070	0.545	0.070
Foliar C/N	Fruit	0.948	8.380	0.910	0.013
Foliar C/N	Mass	24.204	19.470	0.214	0.146
Fruit	Mass	329.645	460.393	0.474	0.084

**B** **N. rustica Variables** Independent >>>	Dependent	Unstandardized estimate	SE	*P*	Standardized estimate
**Direct Effect**					
Foliar C/N	Caterpillars	0.061	0.099	0.533	0.073
Foliar C/N	Sap-suckers	-1.268	0.849	0.135	-0.171
Fruit	Caterpillars	0.003	0.003	0.239	0.149
Fruit	Sap-suckers	0.008	0.022	0.726	0.043
Mass	Caterpillars	0.003	0.006	0.661	0.055
Mass	Sap-suckers	**0.082**	**0.050**	**0.100**	**0.202**
Nitrogen addition	Foliar C/N	0.084	0.514	0.869	0.019
Nitrogen addition	Fruit	22.104	20.538	0.282	0.123
Nitrogen addition	Mass	-8.123	9.377	0.386	-0.101
Rainwater addition	Foliar C/N	0.284	0.513	0.580	0.065
Rainwater addition	Fruit	**34.205**	**20.515**	**0.095**	**0.190**
Rainwater addition	Mass	-0.797	9.366	0.932	-0.010
**Unresolved Covariance/Correlation**					
Caterpillars	Sap-suckers	3.302	3.340	0.323	0.116
Foliar C/N	Fruit	21.371	22.603	0.344	0.111
Foliar C/N	Mass	-7.663	10.295	0.457	-0.087
Fruit	Mass	**1451.595**	**443.932**	**0.001**	**0.414**

Note: Significant ($P \leq 0.05$) and marginally significant ($P \leq 0.10$) results are shown in **bold**.
