# Supplementary material for: Interactive effects of precipitation and nitrogen enrichment on multi-trophic dynamics in plant-arthropod communities
Source: PLoS One. 2018 Aug 2;13(8):e0201219. doi: 10.1371/journal.pone.0201219 (PMC6072000; doi:10.1371/journal.pone.0201219)
Supplement: S5 Table — (PDF) [file pone.0201219.s006.pdf]

S5 Table. Matrices for standardized and unstandardized [in brackets] structural equation model total effects for resource cascades from nitrogen and rainwater additions to herbivores on (A) *Nicotiana tabacum* and (B) *N. rustica*, and corresponding R^2 values for dependent variables.

**A** **N. tabacum**		**Independent Variables**					
		Foliar C/N	Fruit	Mass	Nitrogen addition	Rainwater addition	R^2
**Dependent Variables**	Caterpillars	-0.007 [-0.004]	-0.004 [-0.057]	0.001 [0.021]	-0.171 [-0.027]	-0.047 [-0.007]	0.003
	Foliar C/N				-0.513 [-0.146]	-0.003 [-0.001]	0.021
	Fruit				**49.562 [0.505]**	**20.031 [0.204]**	0.297
	Mass				15.064 [0.077]	**39.376 [0.202]**	0.047
	Sap-suckers	-0.214 [-0.026]	-0.008 [-0.027]	**0.045 [0.309]**	0.394 [0.014]	**1.613 [0.057]**	0.092

**B** **N. rustica**		**Independent Variables**					
		Foliar C/N	Fruit	Mass	Nitrogen addition	Rainwater addition	R^2
**Dependent Variables**	Caterpillars	0.061 [0.073]	0.003 [0.149]	0.003 [0.055]	0.052 [0.014]	0.120 [0.032]	0.039
	Foliar C/N				0.084 [0.019]	0.284 [0.065]	0.005
	Fruit				22.104 [0.123]	**34.205 [0.190]**	0.051
	Mass				-8.123 [-0.101]	-0.797 [-0.010]	0.010
	Sap-suckers	-1.268 [-0.171]	0.008 [0.043]	**0.082 [0.202]**	-0.601 [-0.018]	-0.157 [-0.005]	0.083

Notes: Total effects influenced by significant ($P \leq 0.05$) and marginally significant ($P \leq 0.10$) pathways of direct effects are shown in **bold**. Empty cells in the matrices are NA.
