# Supplementary material for: Interactive effects of precipitation and nitrogen enrichment on multi-trophic dynamics in plant-arthropod communities
Source: PLoS One. 2018 Aug 2;13(8):e0201219. doi: 10.1371/journal.pone.0201219 (PMC6072000; doi:10.1371/journal.pone.0201219)
Supplement: S6 Table — (PDF) [file pone.0201219.s007.pdf]

S6 Table. Standardized and unstandardized structural equation model results for the consumer cascade from spiders to (A) *Nicotiana tabacum* and (B) *N. rustica* traits.

**A** N. tabacum Variables Independent >>>	Dependent	Unstandardized estimate	SE	*P*	Standardized estimate
**Direct Effect**					
Caterpillars	Foliar C	-0.034	0.053	0.517	-0.075
Caterpillars	Foliar N	-0.018	0.035	0.617	-0.058
Caterpillars	Fruit	-0.815	1.787	0.648	-0.053
Caterpillars	Mass	-0.289	3.371	0.932	-0.010
Foliar C	Foliar C/N	**0.159**	**0.039**	**<0.001**	**0.133**
Foliar N	Foliar C/N	**-1.868**	**0.058**	**<0.001**	**-1.035**
Sap-suckers	Foliar C	0.012	0.012	0.313	0.117
Sap-suckers	Foliar N	-0.003	0.008	0.671	-0.049
Sap-suckers	Fruit	0.089	0.404	0.826	0.026
Sap-suckers	Mass	**2.070**	**0.762**	**0.007**	**0.302**
Spider presence	Caterpillars	**-1.211**	**0.734**	**0.099**	**-0.188**
Spider presence	Sap-suckers	-3.749	3.277	0.253	-0.132
**Unresolved Covariance/Correlation**					
Caterpillars	Sap-suckers	2.169	5.182	0.676	0.049
Foliar C	Foliar N	**0.760**	**0.187**	**<0.001**	**0.536**
Foliar C	Fruit	9.325	8.436	0.269	0.130
Foliar C	Mass	-4.190	15.792	0.791	-0.031
Foliar N	Fruit	4.165	5.590	0.456	0.088
Foliar N	Mass	-12.461	10.606	0.240	-0.138
Fruit	Mass	677.843	538.865	0.208	0.149

**B** N. rustica Variables Independent >>>	Dependent	Unstandardized Estimate	SE	*P*	Standardized Estimate
**Direct Effect**					
Caterpillars	Foliar C	-0.227	0.228	0.318	-0.113
Caterpillars	Foliar N	-0.054	0.045	0.227	-0.136
Caterpillars	Fruit	8.231	5.622	0.143	0.169
Caterpillars	Mass	1.706	2.483	0.492	0.079
Foliar C	Foliar C/N	**0.255**	**0.022**	**<0.001**	**0.434**
Foliar N	Foliar C/N	**-3.461**	**0.113**	**<0.001**	**-1.159**
Sap-suckers	Foliar C	**0.063**	**0.026**	**0.015**	**0.275**
Sap-suckers	Foliar N	**0.013**	**0.005**	**0.010**	**0.288**
Sap-suckers	Fruit	0.439	0.638	0.491	0.080
Sap-suckers	Mass	**0.553**	**0.282**	**0.049**	**0.225**
Spider presence	Caterpillars	-0.399	0.431	0.354	-0.108
Spider presence	Sap-suckers	**15.231**	**3.380**	**<0.001**	**0.466**
**Unresolved Covariance/Correlation**					
Caterpillars	Sap-suckers	**5.260**	**3.168**	**0.097**	**0.198**
Foliar C	Foliar N	**1.400**	**0.336**	**<0.001**	**0.559**
Foliar C	Fruit	-1.178	36.885	0.975	-0.004
Foliar C	Mass	**38.789**	**16.914**	**0.022**	**0.279**
Foliar N	Fruit	-6.557	7.277	0.368	-0.106
Foliar N	Mass	4.734	3.244	0.145	0.173
Fruit	Mass	**1267.278**	**428.670**	**0.003**	**0.369**

Note: Significant ($P \leq 0.05$) and marginally significant ($P \leq 0.10$) results are shown in **bold**.
