# Supplementary material for: Interactive effects of precipitation and nitrogen enrichment on multi-trophic dynamics in plant-arthropod communities
Source: PLoS One. 2018 Aug 2;13(8):e0201219. doi: 10.1371/journal.pone.0201219 (PMC6072000; doi:10.1371/journal.pone.0201219)
Supplement: S7 Table — (PDF) [file pone.0201219.s008.pdf]

S7 Table. Matrices for standardized and unstandardized [in brackets] structural equation model total effects for the consumer cascades from spiders to (A) *Nicotiana tabacum* and (B) *N. rustica* traits, and corresponding R^2 values for dependent variables.

**A** N. tabacum		Independent Variables					
		Caterpillars	Foliar C	Foliar N	Sap-suckers	Spider presence	**R^2**
**Dependent Variables**	Caterpillars					**-1.211 [-0.188]**	0.035
	Foliar C	-0.034 [-0.075]			0.012 [0.117]	-0.004 [-0.001]	0.018
	Foliar N	-0.018 [-0.058]			-0.003 [-0.049]	0.034 [0.017]	0.006
	Foliar C/N	0.027 [0.050]	**0.159 [0.133]**	**-1.868 [-1.035]**	0.008 [0.067]	-0.064 [-0.018]	0.944
	Fruit	-0.815 [-0.053]			0.089 [0.026]	0.653 [0.007]	0.003
	Mass	-0.289 [-0.010]			**2.070 [0.302]**	-7.410 [-0.038]	0.091
	Sap-suckers					-3.749 [-0.132]	0.017

**B** N. rustica		Independent Variables					
		Caterpillars	Foliar C	Foliar N	Sap-suckers	Spider presence	**R^2**
**Dependent Variables**	Caterpillars					-0.399 [-0.108]	0.012
	Foliar C	-0.227 [-0.113]			**0.063 [0.275]**	**1.048 [0.141]**	0.081
	Foliar N	-0.054 [-0.136]			**0.013 [0.288]**	**0.219 [0.149]**	0.092
	Foliar C/N	0.129 [0.108]	**0.255 [0.434]**	**-3.461 [-1.159]**	**-0.029 [-0.215]**	**-0.492 [-0.112]**	0.932
	Fruit	8.231 [0.169]			0.439 [0.080]	3.402 [0.019]	0.038
	Mass	1.706 [0.079]			**0.553 [0.225]**	**7.748 [0.096]**	0.061
	Sap-suckers					**15.231 [0.466]**	0.218

Notes: Total effects influenced by significant ($P \leq 0.05$) and marginally significant ($P \leq 0.10$) pathways of direct effects are shown in **bold**. Empty cells in the matrices are NA.
