# Supplementary figures and images for: Interactive effects of precipitation and nitrogen enrichment on multi-trophic dynamics in plant-arthropod communities
Source: PLoS One. 2018 Aug 2;13(8):e0201219. doi: 10.1371/journal.pone.0201219 (PMC6072000; doi:10.1371/journal.pone.0201219)

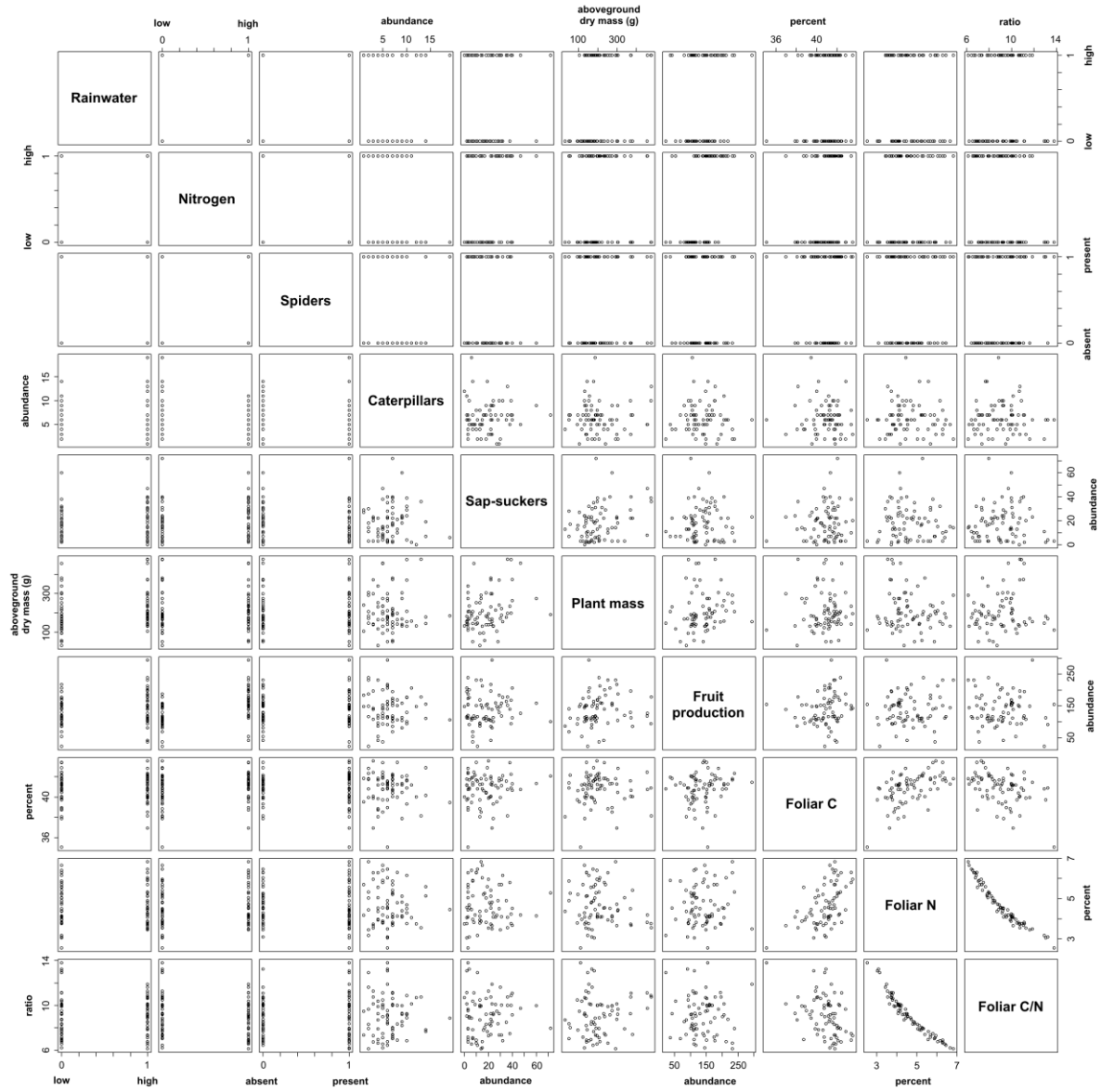

**S1 Fig. Bivariate scatterplots for variables used in the *N. tabacum* structural equation models.**

Supplement: S1 Fig — (PDF) [file pone.0201219.s009.pdf]

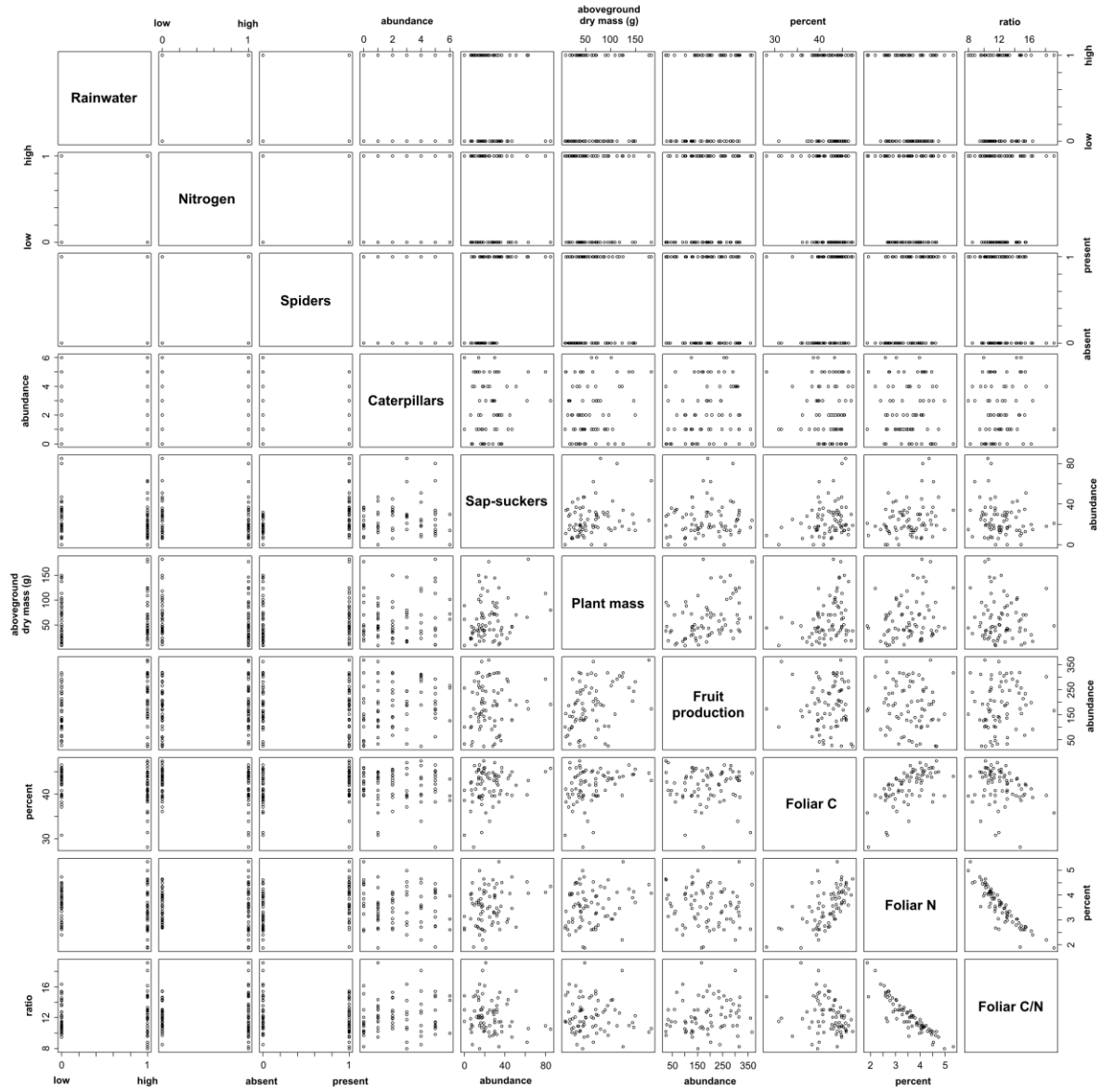

**S1 Fig. Bivariate scatterplots for variables used in the *N. rustica* structural equation models.**

Supplement: S2 Fig — (PDF) [file pone.0201219.s010.pdf]
